# Optimized Dropkey-Based Grad-CAM: Toward Accurate Image Feature Localization

**DOI:** 10.3390/s23208351

**Published:** 2023-10-10

**Authors:** Yiwei Liu, Luping Tang, Chen Liao, Chun Zhang, Yingqing Guo, Yixuan Xia, Yangyang Zhang, Sisi Yao

**Affiliations:** 1 College of Mechanical and Electrical Engineering, Nanjing Forestry University, Nanjing 210037, China; 2 SEU-FEI Nano-Pico Center, Key Lab of MEMS of Ministry of Education, Southeast University, Nanjing 210096, China; 3 College of Electronic and Optical Engineering & College of Flexible Electronics (Future Technology), Nanjing University of Posts and Telecommunications, Nanjing 210023, China; chenliao@njupt.edu.cn

**Keywords:** convolutional neural networks, class activation mapping, interpretability, computer vision

## Abstract

Regarding the interpretable techniques in the field of image recognition, Grad-CAM is widely used for feature localization in images to reflect the logical decision-making information behind the neural network due to its high applicability. However, extensive experimentation on a customized dataset revealed that the deep convolutional neural network (CNN) model based on Gradient-weighted Class Activation Mapping (Grad-CAM) technology cannot effectively resist the interference of large-scale noise. In this article, an optimization of the deep CNN model was proposed by incorporating the Dropkey and Dropout (as a comparison) algorithm. Compared with Grad-CAM, the improved Grad-CAM based on Dropkey applies an attention mechanism to the feature map before calculating the gradient, which can introduce randomness and eliminate some areas by applying a mask to the attention score. Experimental results show that the optimized Grad-CAM deep CNN model based on the Dropkey algorithm can effectively resist large-scale noise interference and achieve accurate localization of image features. For instance, under the interference of a noise variance of 0.6, the Dropkey-enhanced ResNet50 model achieves a confidence level of 0.878 in predicting results, while the other two models exhibit confidence levels of 0.766 and 0.481, respectively. Moreover, it exhibits excellent performance in visualizing tasks related to image features such as distortion, low contrast, and small object characteristics. Furthermore, it has promising prospects in practical computer vision applications. For instance, in the field of autonomous driving, it can assist in verifying whether deep learning models accurately understand and process crucial objects, road signs, pedestrians, or other elements in the environment.

## 1. Introduction

Convolutional neural network (CNN) models have been widely applied in the field of computer vision, such as object detection [1,2] and image classification [3,4], due to their remarkable feature extraction capabilities. However, the complex network structure, parameter sharing, and non-linear operations of CNN models pose challenges in effectively interpreting their output [5,6]. Furthermore, in real-world scenarios, the input images or videos are often non-standard [7]. They are frequently influenced by environmental factors, camera variations, image distortions, and other such factors. These complexities pose challenges to the research on model interpretability. However, despite these difficulties, it remains crucial to address the interpretability of models in order to ensure their effectiveness and reliability in practical applications. Through continued advancements in techniques such as Dropkey Grad-CAM (Gradient-weighted Class Activation Mapping), we can strive to overcome these challenges and enhance the interpretability of models even in the face of real-world complexities. Various methods have been proposed to enhance the interpretability of CNN models, which can be categorized into perturbation-based [8,9,10], propagation-based [11,12], and activation-based approaches. Among the activation-based approaches, Class Activation Maps (CAM) [13,14] have gained significant popularity due to their ability to provide intuitive visualizations for CNNs.

CAM techniques have limitations in practical usage as they are specific to certain architectures and require architectural modifications. To address this, Selvaraju et al. proposed Grad-CAM, which allows visualization and interpretation of CNN models without architectural changes [15]. Further improvements include Smooth Grad-CAM, which reduces noise and uncertainty in visualization results [16], and Score-CAM, which enables high-resolution activation map generation [17]. These advancements enhance the understanding and application of CNNs in computer-aided diagnosis.

Due to the nature of Grad-CAM, which expresses the decision information of the model in the form of a heatmap, we can indirectly improve the robustness of Grad-CAM by enhancing the model’s robustness. There are six methods to improve model robustness: Data Augmentation, Regularization, Ensemble Learning, Adversarial Training, Weight Pruning, and Reinforcement Learning. In the field of Data Augmentation, Ekin D. Cubuk et al. proposed a method called AutoAugment [18], which enhances the model’s adaptability to different samples. This augmentation helps the model learn more robust feature representations, thereby improving the model’s robustness when facing perturbations. In the field of Adversarial Training, Alexey Kurakin et al. introduced adversarial examples in the physical world and found that certain physical-world samples can highly confuse the model [19]. This discovery has inspired further improvements in model robustness. In the field of Regularization, Hongyi Zhang et al. proposed a method called Mixup [20], which performs linear interpolation between two different samples to create new training samples. This approach provides smoother training information, leading to improved generalization ability and robustness of neural network models. These methods, along with others such as Ensemble Learning, Weight Pruning, and Reinforcement Learning, contribute to enhancing the robustness of models in various ways, which can indirectly improve the robustness of Grad-CAM as well.

In practical applications, images are often subjected to various disturbances, such as rotation, translation, noise, and changes in lighting conditions. Therefore, the system needs to tolerate such disturbances while maintaining its original output. However, Grad-CAM primarily relies on gradient information, which makes it ineffective in resisting large-scale noise interference [21]. In this study, the Dropkey algorithm [22] is employed to optimize CNN models and improve the robustness of Grad-CAM. The optimization process involves passing the input through the convolutional network to extract output features from a specified layer (layer 4). These features undergo pooling and are compressed into a one-dimensional representation. Subsequently, the resulting output serves as the Q (Query), K (Key), and V (Value) parameters in the Attention function, which generates the attention map. Finally, the attention map is passed through a fully connected layer for classification, resulting in predicted outcomes. To evaluate the performance of Grad-CAM and the optimized Dropkey-based Grad-CAM in the presence of varying levels of noise interference, Gaussian noise with different variances is added to images to simulate real-world disturbances. The findings indicate that the optimized Dropkey-based Grad-CAM effectively resists large-scale noise perturbations. In addition, the optimized Dropkey-based Grad-CAM demonstrates superior performance in various testing tasks, including feature localization in low-contrast images and feature localization of small objects. This technology also holds promising prospects in various real-life applications. For instance, in the field of autonomous driving, Dropkey Grad-CAM can be employed in deep learning models to provide an explainable analysis of the decision-making process of autonomous driving systems, ensuring their safety and reliability. By utilizing Dropkey Grad-CAM, we can interpret the model’s rationale for behavior prediction and visualize the areas of focus in the traffic scene by the deep learning model. This approach enhances transparency and allows for a comprehensive understanding of how the model arrives at its decisions, ultimately contributing to the trustworthiness and interpretability of autonomous driving systems.

## 2. Approach

### 2.1. Working Principle of Grad-CAM

Grad-CAM can be comprehensively explained through mathematical derivation. By differentiating the output $y^{c}$ of category c with respect to the feature map of the convolution layer, the gradient information for each category in the image can be obtained as $\partial y^{c}/\partial A^{k}$, where $A^{k}$ represents the class activation mapping of Grad-CAM. Back-propagating all the gradient information leads to Equation (1) [15]. For the prediction result vector y, $y^{c}$ represents the score of the target class c, which can be obtained through the output of the last fully connected layer or softmax layer of the network, depending on the network architecture and task.

(1) $$\alpha_{k}^{c}=\frac{1}{Z}\sum_{i}\sum_{j}\;\frac{\partial y^{c}}{\partial A_{ij}^{k}}$$

In the equation, $\alpha_{k}^{c}$ denotes the linearization coefficient of the downstream part of the neural network model, which also represents the weight information of feature map k for a specific class c. Z denotes the number of channels.

(2) $$L^{c}=\mathrm{ReLU}\left(\sum_{k}\;\alpha_{k}^{c}A^{k}\right)$$

By tracking the weighted combination of forward-activated maps using the ReLU activation function, a heatmap that aligns with the size of the convolutional feature maps can be obtained, as shown in Equation (2) [15].

Grad-CAM computes the feature maps of the output layer and computes gradients of the respective categories by leveraging input information. Subsequently, the gradients associated with each category are weighted and averaged across channels, and the positive weights for each channel are aggregated. Consequently, a heat map is generated using these calculations. Each pixel within the heat map conveys its weight representation pertaining to the designated category [9]. The heat map is derived by selectively considering the positive weights from each channel and aggregating them collectively.

### 2.2. Improvement of Grad-CAM Using Dropout-Based Method

When training a CNN model, it is crucial to address the issue of overfitting, especially when the model has a large number of parameters and the training dataset is limited in size [23]. In 2012, Hinton introduced the Dropout algorithm, which aims to mitigate overfitting in complex feedforward neural networks with small training datasets [24]. Grad-CAM calculates a weight matrix by computing gradients with respect to the feature map, which is subsequently used to generate a heatmap depicting the regions of interest for a given model. However, most deep learning models exhibit high nonlinearity and complexity, leading to potential influences of specific connections within the model on the generated Grad-CAM heatmaps. Consequently, the robustness of visual interpretability results might be compromised. In order to mitigate this issue, Dropout can be introduced during the computation process to significantly reduce the reliance on specific neurons. Specifically, Dropout involves randomly discarding certain neurons, which forces the model to learn from an ensemble of sub-models. Each sub-model possesses random variations in feature representations. Consequently, by employing this approach, the generated heatmap considers a wider range of neuron combinations, thus reducing excessive dependence on the activation of any particular neuron combination. Introducing Dropout in this manner enhances the robustness of the generated heatmaps, enabling them to more reliably reflect the regions of interest identified by the model. This approach mitigates potential biases and noise, providing a more comprehensive representation of the model’s focus on the target class without overemphasizing specific neurons or connections. Consequently, we can more reliably determine the basis for the model’s decisions.

### 2.3. Improvement of Grad-CAM Using Dropkey-Based Method

Dropkey is an innovative Dropout method inspired by DropAttention. It adapts attention weights in neural networks to promote smoother attention vectors [22,25]. Dropkey employs a Dropout-before-softmax approach and migrates the Dropout operation before attention matrix calculation, using the key unit as the new Dropout unit. Unlike the constant drop rate of Dropout, Dropkey gradually decreases the drop rate along the self-attention layers. This approach ensures system stability, facilitates attention weight regularization, and mitigates the loss of high-level semantics and overfitting.

During neural network training, Dropkey randomly discards a percentage of the input key units, generating an independent mask key mapping for each query. This masking of the attention scoring matrix positions Dropkey as a technique that modifies the placement of Dropout, lagging it to mitigate model overfitting. The mathematical formulation of Dropkey can be analyzed using Equations (3) and (4).

(3) $$p_{j}=\frac{\exp\left(d_{j}+\frac{qk_{j}^{T}}{scale}\right)}{\sum_{j=1}^{n_{h}n_{w}}\;\exp\left(d_{j}+\frac{qk_{j}^{T}}{scale}\right)}$$

(4) $$o=\sum_{j=1}^{n_{h}n_{w}}\;p_{j}v_{j}$$

In the equations, $p_{j}$ represents the attention weights in the attention mechanism, o denotes the output patch, $q_{i}$ denotes the i-th patch of the query, $k_{j}$ denotes the key unit of the j-th patch of the query, $v_{j}$ denotes the value of the j-th patch of the query. Suppose the input image contains three types of information: height, width, and number of channels; the image can be divided into blocks of $n_{h}$ ∗ $n_{w}$. The expression $n_{h}$ ∗ $n_{w}$ represents the number of blocks into which the image is divided along the height and width directions, respectively. Here, $n_{h}$ represents the number of blocks along the height direction and $n_{w}$ represents the number of blocks along the width direction. The scale represents the scaling factor. 

**Figure 1:**
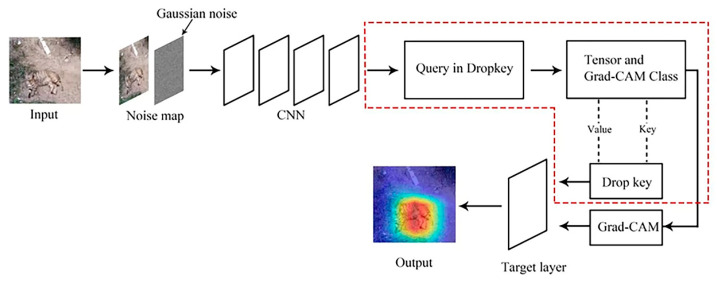
The refined design approach for Grad-CAM based on Dropkey.

The optimized Dropkey-based Grad-CAM framework is illustrated in Figure 1. The algorithm begins by passing the input through a convolutional network and extracting the output features from the specified layer (layer 4). The features are then passed through a pooling layer for pooling and compressed into a one-dimensional form. The resulting output serves as the parameters Q, K, and V, which are fed into the Attention function to obtain the attention map. Finally, the attention map is passed to a fully connected layer for classification, resulting in the prediction.

Overview of optimized Dropkey-based Grad-CAM: The Dropkey algorithm introduces a Bernoulli noise layer on the attention score matrix, which randomly masks a certain proportion of attention weights and suppresses unnecessary signals. This prevents the neural network model from overly relying on a piece of specific local information. Then, Dropkey applies the softmax operation along the last dimension of the attention score matrix to obtain weight coefficients. These coefficients are used to weigh the value vectors and compute their average. Finally, the weighted average is returned as the encoded representation of the query vector, q. The content within the dashed box describes the functioning mechanism of the Q, K, and V parameters in Dropkey.

Introducing the Dropkey attention mechanism into Grad-CAM can reduce the reliance on specific neurons during the calculation process by incorporating random binary masks. Specifically, when computing attention weights, a binary random mask tensor of the same shape as the original attention weights are multiplied element-wise with the attention weights. Additionally, a small negative value is multiplied to set the attention weights to negative infinity at the masked positions. This ensures that the attention weights at the masked positions, after the softmax operation, are effectively zero, thus reducing their contribution to the final visual interpretability results. Furthermore, the Dropkey mechanism introduces randomness and enhances the generalization capability of the visual interpretability results by randomly discarding a portion of neurons. Through this mechanism, Grad-CAM becomes more adaptable to different input samples, thereby improving the generalization of the results. Consequently, the generated heatmaps better reflect the model’s attention to the target feature regions in a more comprehensive manner. Compared to Grad-CAM, improved with Dropout, the Dropkey mechanism alters the distribution of attention weights by introducing random masks and neuron Dropout. This enhances the randomness of the attention weights, leading to heat maps that are more interpretable and stable. Overall, the Dropkey-enhanced Grad-CAM exhibits superior robustness and generalization capability. This allows for a more accurate understanding of the model’s focus on input image features and the decision-making process. It contributes to improving the accuracy, reliability, and interpretability of explaining model predictions.

## 3. Result and Discussion

### 3.1. Robustness of Grad-CAM and Optimized Dropkey-Based Grad-CAM

**Figure 2:**
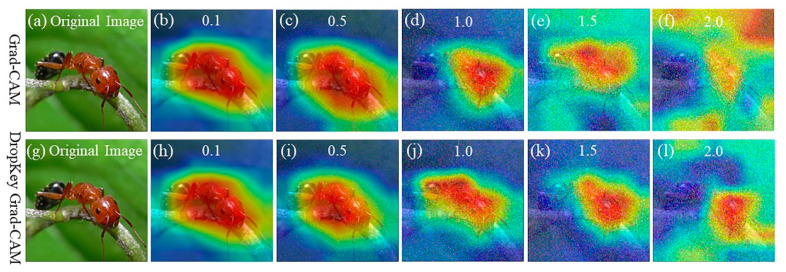
The visualization of Grad-CAM and Dropkey Grad-CAM by adding noise with variances of 0.1, 0.5, 1.0, 1.5, and 2.0 to images. It is clear that both of these two approaches are able to highlight the object of interest under a variance below 1, but Dropkey Grad-CAM provides more details than Grad-CAM under a variance of 1. Note that red regions correspond to high scores for class. Figure best viewed in color.

The robustness of the optimized Dropkey-based Grad-CAM technique is evaluated in this section. Grad-CAM, relying on gradient information, may face challenges in accurately localizing image features when the input images are subjected to various disturbances such as blurriness or image noise. To assess the performance of the proposed method under different noise conditions, input images were augmented with noise of varying variances, specifically 0.1, 0.5, 1.0, 1.5, and 2.0. It is important to note that the pixel values of the augmented images were normalized to ensure they remained within the valid range of 0 to 255. The visualizations obtained from both Grad-CAM and the optimized Dropkey-based Grad-CAM techniques under the different noise conditions are presented in Figure 2.

As shown in Figure 2, it can be clearly observed that Grad-CAM maintains good accuracy in classifying the specified category when the variance of the added noise is below 0.5 (Figure 2b,c), while the accuracy of Grad-CAM classification significantly decreases when the noise variance changes from 1.0 to 1.5 (Figure 2d,e). As the noise variance gradually increases, Grad-CAM loses its resistance to noise, eventually leading to misclassifications (Figure 2f). Therefore, it can be concluded that Grad-CAM exhibits good accuracy in the presence of noise within a certain range, ensuring the normal and stable operation of the neural network model. However, when the noise exceeds a certain range, Grad-CAM cannot guarantee the proper functioning of the system, fails to locate the specified feature information in the image, and may even result in classification errors. Therefore, robustness optimization is necessary for Grad-CAM.

On the other hand, Grad-CAM improved with the Dropkey algorithm consistently demonstrates superior localization performance when the noise variance is 1.0 or below (Figure 2h–j). It is evident that both Grad-CAM and the optimized Dropkey-based Grad-CAM effectively highlight the object of interest under a variance below 1. However, the optimized Dropkey-based Grad-CAM provides more detailed visualizations compared to Grad-CAM under a variance of 1 (Figure 2d,j). Remarkably, even in the presence of noise with a variance of 2.0, the improved Grad-CAM based on Dropkey does not exhibit misclassifications, as depicted in Figure 2l. With gradually increasing Gaussian noise, the optimized Dropkey-based Grad-CAM visualization method demonstrates excellent robustness, preserving the stability of visualization results and accurately highlighting the featured objects in the image. In contrast, Grad-CAM exhibits noticeable degradation effects under the same noise interference, resulting in visual distortions and uninterpretable outcomes. Moreover, the optimized Dropkey-based Grad-CAM can be applied to most models, making it crucial for interpreting the logical decision information of computer vision models and improving their interpretability.

**Figure 3:**
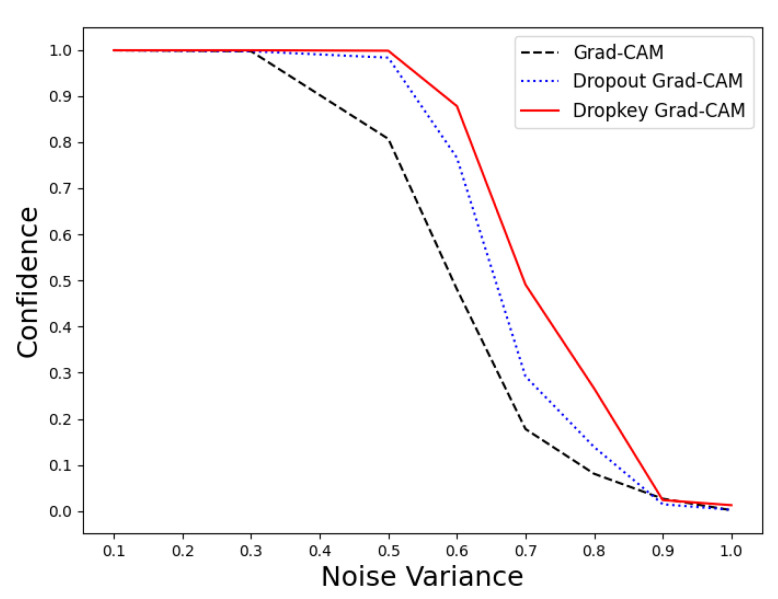
The confidence level variations of three models in image classification tasks under noise interference.

Due to its inherent nature as an improvement to the ResNet50 model, we can indirectly observe the resilience of Dropkey Grad-CAM to noise by examining the changes in the model’s confidence in prediction results under the influence of Gaussian noise of varying variances. The experimental results are illustrated in Figure 3.

From the data in the graph, it can be observed that when the Gaussian noise variance is below 0.3, all three methods, namely Grad-CAM, Dropout Grad-CAM, and Dropkey Grad-CAM, demonstrate good resistance and maintain a high level of confidence. However, when the Gaussian noise variance reaches 0.5, Grad-CAM’s resistance to noise diminishes. Furthermore, during the transition from a variance of 0.5 to 0.9, all three methods experienced a decrease in confidence levels. Nevertheless, from the data in the graph, it is evident that Dropkey Grad-CAM exhibits better confidence levels compared to the other two methods under the influence of noise of the same magnitude.

Under the interference of the same noise, the ResNet50 model improved with the Dropkey mechanism and demonstrated superior confidence in prediction results. For instance, when the noise variance is 0.5, the confidence levels for the prediction results of the Dropkey-enhanced ResNet50 model and the Dropout-enhanced ResNet50 model are 0.998 and 0.983, respectively, while the confidence level for the original ResNet50 model drops to 0.807. Furthermore, under the interference of a noise variance of 0.6, the Dropkey-enhanced ResNet50 model maintains a confidence level of 0.878, while the Dropout-enhanced ResNet50 model and the original ResNet50 model experience significant drops in confidence levels to 0.766 and 0.481, respectively. It is evident from the model analysis that the Dropkey-enhanced ResNet50 model possesses better noise resistance capabilities. Additionally, improvements in the model contribute to the enhancement of Grad-CAM performance. Therefore, we can indirectly conclude that the introduction of the Dropkey mechanism enables effective mitigation of noise interference, thereby improving the accuracy of Grad-CAM.

### 3.2. Distorted Image Feature Localization

**Figure 4:**
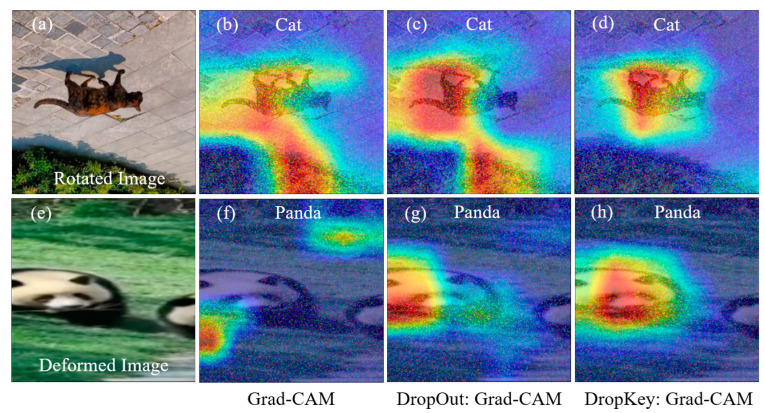
Robustness analysis of the optimized Dropout-based Grad-CAM, Grad-CAM, and optimized Dropkey-based Grad-CAM for localizing rotated features (**a**–**d**) and deformed features (**e**–**h**) in images.

Furthermore, changes in the structure and shape of an image, such as rotation or stretching deformation, can also impact the localization of image features. Image rotation alters the position and orientation of image features, potentially affecting the accuracy of feature localization. Similarly, stretching deformation modifies the aspect ratio of target features, leading to errors in feature localization and weight calculation, thereby influencing the accuracy of the obtained results. As illustrated in Figure 4, both Grad-CAM (Figure 4b) and the improved Grad-CAM based on Dropout (Figure 4c) can locate the target object in the rotated image. However, the positioning of the target object is not sufficiently precise, and classification errors may occur. In contrast, the improved Grad-CAM based on Dropkey can accurately locate target features without any classification errors (Figure 4d). It is worth noting that Grad-CAM fails to locate target features in the stretched image (Figure 4f). Although the improved Grad-CAM based on Dropout can locate target features, its accuracy is compromised, resulting in the loss of edge features (Figure 4g). Conversely, the improved Grad-CAM based on Dropkey can effectively locate target features, delivering more precise positioning results without sacrificing edge features (Figure 4h).

**Figure 5:**
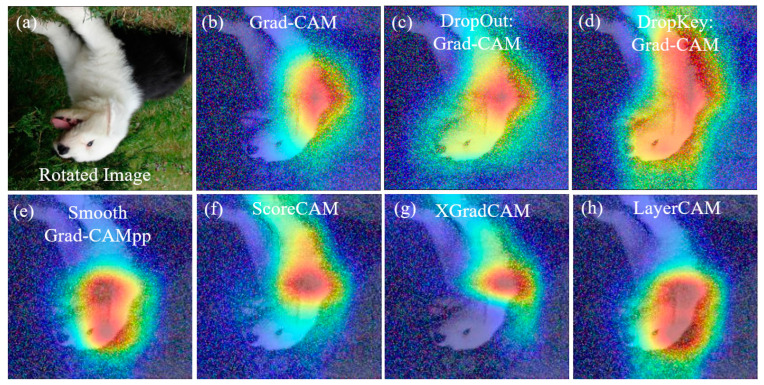
Under noise interference with a variance of 1.5, the comparison between Dropkey Grad-CAM (**d**) and mainstream methods in the field of visual explanation ((**a**–**c**) and (**e**–**h**)).

To further validate the resistance performance of Dropkey-enhanced Grad-CAM against large-scale noise, we selected four mainstream methods in the field of visual explanation for comparative verification (Figure 5).

The experimental results indicate that under noise interference with a variance of 1.5, both Grad-CAM and its four variant methods fail to accurately locate the entire feature object. They only make judgments based on certain parts of the feature target. On the other hand, Grad-CAM optimized with Dropout and Dropkey can relatively accurately locate the target feature, and Dropkey Grad-CAM exhibits more complete feature localization. This further demonstrates the excellent performance of Dropkey Grad-CAM in resisting noise interference.

### 3.3. Low-Contrast Image Feature Localization

The localization of low-contrast image features is often challenging due to the absence of significant brightness variations and distinct edges, resulting in blurred or ambiguous details within the image. Additionally, noise in low-contrast images tends to be more prominent, causing the target features to be submerged or mixed, thereby greatly impacting extraction and localization. Consequently, the recognition and extraction of low-contrast image features present a formidable task in assessing computer visualization and interpretability.

**Figure 6:**
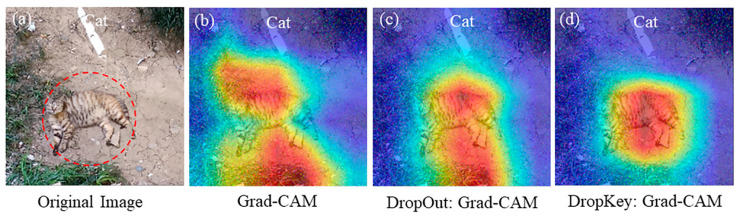
Visualization of optimized Dropkey-based Grad-CAM for localizing small features in images (**d**).

Based on experimental results, it is evident that the original Grad-CAM technique exhibits significant distortions when extracting low-contrast image features, rendering it incapable of effectively locating the target feature objects in the images, as illustrated in Figure 6b. The improved Grad-CAM technique based on Dropout can mitigate some of the noise interference compared to the original Grad-CAM. However, it suffers from the loss of edge information pertaining to the target features, leading to interpretability errors, as shown in Figure 6c. In contrast, the improved Grad-CAM based on Dropkey technology can precisely and clearly locate the key feature objects in low-contrast images. Moreover, it demonstrates superior visualization results in terms of feature edge and texture details, thereby effectively reducing the influence of noise and enhancing the system’s robustness under low-contrast conditions, as shown in Figure 6d.

### 3.4. Image Feature Localization of Extremely Small Objects

The recognition and extraction of extremely small feature objects pose a significant challenge in computer visualization. Due to resolution limitations, pictures containing small feature objects often suffer from detail loss, which hampers accurate extraction and identification. Moreover, noise can substantially affect the recognition, extraction, and matching of smaller features.

**Figure 7:**
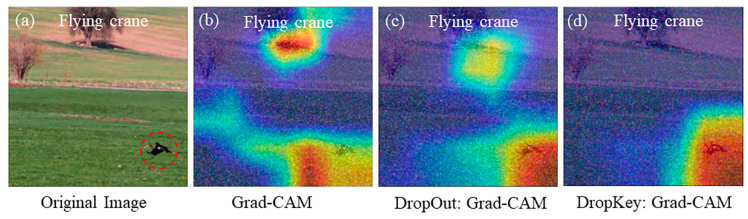
Visualization of optimized Dropkey-based Grad-CAM for localization of small features in images (**d**).

Based on the experimental results, it is evident that the original Grad-CAM technique fails to effectively locate small target feature objects in the presence of noise interference (Figure 7b). Although the improved Grad-CAM based on Dropout can locate the target feature objects, it lacks accuracy and still exhibits recognition errors, as depicted in Figure 7c. In contrast, the improved Grad-CAM based on Dropkey achieves precise localization of small feature objects, mitigating image classification errors and outperforming the Grad-CAM in terms of accuracy. This robust and reliable performance of the improved Grad-CAM based on Dropkey is demonstrated in small feature recognition tasks, affirming its adaptability in real-world environments, as illustrated in Figure 7d.

**Figure 8:**
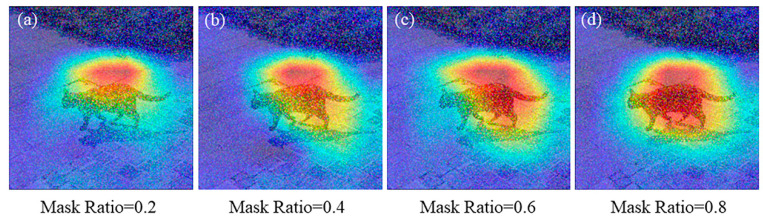
Evaluation of the robustness performance of Dropkey Grad-CAM using different masking ratios on images.

Additionally, it is important to note that in practical usage, we should pay attention to the selection of the mask ratio parameter and choose an appropriate value based on different input scenarios. For example, in the following experiment, the visualization heatmaps may vary depending on the chosen mask ratio value (Figure 8).

This phenomenon arises because a smaller mask ratio implies fewer lines are “removed” from the model as the proportion of discarded keywords in the Dropkey algorithm decreases. Conversely, a larger mask ratio corresponds to more lines being “removed” from the model as the proportion of discarded keywords in the Dropkey mechanism increases. Consequently, the mask ratio and the number of rows removed or retained in Dropkey exhibit a proportional relationship.

In simpler terms, when the mask ratio approaches 0, the Dropkey mechanism has minimal impact, meaning that only a few rows are discarded. On the other hand, when the mask ratio approaches 1, the Dropkey mechanism becomes highly influential, leading to the removal of more rows. To put it simply, when the influence of Dropkey is low, there is a higher risk of overfitting the model. On the other hand, when the influence of Dropkey is high, it can negatively impact the predictive performance of the model. Hence, selecting an appropriate mask ratio becomes crucial, considering the specific circumstances and striking a proper balance between model prediction performance and overfitting.

## 4. Conclusions

In this paper, the robustness of Grad-CAM is tested and improved. Since Grad-CAM heavily relies on gradient calculations, it is susceptible to interference information. To ensure its stability and effectiveness, Dropout and Dropkey algorithms are employed to improve its robustness. Experimental results demonstrate that both Dropout and Dropkey algorithms improve the generalization capability of the CNN model, thereby improving the robustness of the Grad-CAM technique. During the experiments, it was observed that the selection of the mask ratio parameter in the Dropkey algorithm significantly influences the final Grad-CAM localization effect. Therefore, it is crucial to carefully choose the appropriate parameter under specific experimental conditions and image characteristics. Furthermore, the experimental findings indicate that the improved Grad-CAM based on the Dropkey algorithm exhibits greater robustness compared to the enhanced Grad-CAM based on Dropout. This can help people better validate whether neural network models can correctly process information in images.

## Data Availability

Data is unavailable due to privacy restrictions.
